# Advanced neoplasia in Veterans at screening colonoscopy using the National Cancer Institute Risk Assessment Tool

**DOI:** 10.1186/s12885-019-6204-1

**Published:** 2019-11-12

**Authors:** Laura W. Musselwhite, Thomas S. Redding, Kellie J. Sims, Meghan C. O’Leary, Elizabeth R. Hauser, Terry Hyslop, Ziad F. Gellad, Brian A. Sullivan, David Lieberman, Dawn Provenzale

**Affiliations:** 1 VA Cooperative Studies Program Epidemiology Center, Durham Veterans Affairs Health Care System, 508 Fulton Street, Durham, NC 27705 USA; 2 Levine Cancer Institute, Atrium Health, 100 Medical Park Drive, Suite 110 Concord, Charlotte, NC 28025 USA; 3 Duke Molecular Physiology Institute, Duke University Medical Center, Durham, NC USA; 4 Duke University Medical Center, Duke University, 2424 Erwin Road, 8037 Hock Plaza, Durham, NC 27705 USA; 5 Department of Medicine, Duke University School of Medicine, Durham, NC USA; 6 Veterans Affairs Portland Health Care System, 3710 Sw US Veterans Hospital Road, Portland, OR 97239 USA; 7 Oregon Health & Science University, 3181 Sw Sam Jackson Park Road, Portland, OR 97239 USA

**Keywords:** Colorectal advanced neoplasia, Colorectal cancer screening, Veteran, Screening colonoscopy, Risk assessment

## Abstract

**Background:**

Adapting screening strategy to colorectal cancer (CRC) risk may improve efficiency for all stakeholders however limited tools for such risk stratification exist. Colorectal cancers usually evolve from advanced neoplasms that are present for years. We applied the National Cancer Institute (NCI) CRC Risk Assessment Tool, which calculates future risk of CRC, to determine whether it could be used to predict current advanced neoplasia (AN) in a veteran cohort undergoing a baseline screening colonoscopy.

**Methods:**

This was a prospective assessment of the relationship between future CRC risk predicted by the NCI tool, and the presence of AN at screening colonoscopy. Family, medical, dietary and physical activity histories were collected at the time of screening colonoscopy and used to calculate absolute CRC risk at 5, 10 and 20 years. Discriminatory accuracy was assessed.

**Results:**

Of 3121 veterans undergoing screening colonoscopy, 94% had complete data available to calculate risk (*N* = 2934, median age 63 years, 100% men, and 15% minorities). Prevalence of AN at baseline screening colonoscopy was 11 % (*N* = 313). For tertiles of estimated absolute CRC risk at 5 years, AN prevalences were 6.54% (95% CI, 4.99, 8.09), 11.26% (95% CI, 9.28-13.24), and 14.21% (95% CI, 12.02-16.40). For tertiles of estimated risk at 10 years, the prevalences were 6.34% (95% CI, 4.81-7.87), 11.25% (95% CI, 9.27-13.23), and 14.42% (95% CI, 12.22-16.62). For tertiles of estimated absolute CRC risk at 20 years, current AN prevalences were 7.54% (95% CI, 5.75-9.33), 10.53% (95% CI, 8.45-12.61), and 12.44% (95% CI, 10.2-14.68). The area under the curve for predicting current AN was 0.60 (95% CI; 0.57-0.63, *p* < 0.0001) at 5 years, 0.60 (95% CI, 0.57-0.63, *p* < 0.0001) at 10 years and 0.58 (95% CI, 0.54-0.61, *p* < 0.0001) at 20 years.

**Conclusion:**

The NCI tool had modest discriminatory function for estimating the presence of current advanced neoplasia in veterans undergoing a first screening colonoscopy. These findings are comparable to other clinically utilized cancer risk prediction models and may be used to inform the benefit-risk assessment of screening, particularly for patients with competing comorbidities and lower risk, for whom a non-invasive screening approach is preferred.

## Background

Colorectal cancer (CRC) screening is a cost-effective [1] and lifesaving strategy [2] for cancer prevention and control. However, only a small minority of patients will derive direct individual benefit and others may receive a false positive screening result, prompting invasive procedures that may cause serious adverse events [3]. At the health system level, blanket screening approaches can strain fragile health care systems with limited infrastructures to implement screening programs [4].

In the era of personalized medicine, precision cancer screening aims to risk stratify asymptomatic individuals through the use of patient-specific factors to determine those who are likely and unlikely to benefit from screening.

The National Cancer Institute (NCI) CRC Risk Assessment Tool was developed as a decision-making adjunct in 2009 using U.S.-based case-control studies for colon and rectal cancer and Surveillance and Epidemiology and End Results (SEER) Program data [5]. The model estimates the absolute risk that an individual will develop CRC using well-established clinical risk factors including age, history of colonoscopy or endoscopy in the last 10 years and whether polyps were observed, family history of CRC, weekly physical activity, aspirin or non-steroidal anti-inflammatory drug (NSAID) use, smoking, vegetable intake, and body mass index (BMI). Park et al. externally validated the model in white men and women from a natural history cohort and observed modest discriminatory accuracy and good calibration [6].

Defining the model’s performance as it pertains to predicting CRC precursors provides an opportunity to assess whether the NCI tool can be used to inform patient-provider decision-making on CRC screening. While recent studies have shown that the NCI tool is predictive of advanced neoplasia (AN) in individuals undergoing screening and surveillance colonoscopy [7–9], these studies have not included U.S. veterans, many of whom have unique environmental exposures [10] and cancer risk profiles [11], not fully described or included in prior studies. To inform current CRC prevention strategies within the Veterans Health Administration, which currently cares for *9 million Veterans*, our primary study objective was to externally validate the NCI tool for current advanced neoplasia in a veteran cohort undergoing first screening colonoscopy.

## Methods

### Risk assessment tool

In this prospective study, we evaluated whether the NCI tool, which predicts future CRC risk at 5, 10 and 20 years, could assess current AN risk at the time of baseline screening colonoscopy in the CSP #380 veterans cohort. Variables, classification, and the model included in the NCI CRC Risk Assessment Tool have been published previously [5]. The NCI tool and SAS code are publicly available on the website https://www.cancer.gov/colorectalcancerrisk/ to estimate individual absolute CRC risk over time. We used this tool to calculate 5 10 and 20 year absolute CRC risk and applied resulting risk estimates to model current AN at baseline colonoscopy.

### Study participants

Our study was conducted using the CSP #380 veterans cohort. Approximately 3121 asymptomatic veterans from 13 diverse VA Medical Centers between the ages of 50-75 years were recruited to assess the role of screening colonoscopy between 1994 and 1997. Exclusion criteria included active gastrointestinal disease, lower endoscopy in the previous 10 years, colon surgery, significant co-morbidity, or other medical condition that would increase the risk of performing a screening colonoscopy [12].

At enrollment, a validated, detailed questionnaire on medical history and lifestyle factors was administered and subsequently a baseline screening colonoscopy was performed within 6 months of questionnaire completion. The cohort is made up of 15% minorities and 95% men, reflecting the make-up of the U.S. veteran population in the 1990s. Further information about detailed questionnaires and disease confirmation is published elsewhere [12].

**Fig. 1:**
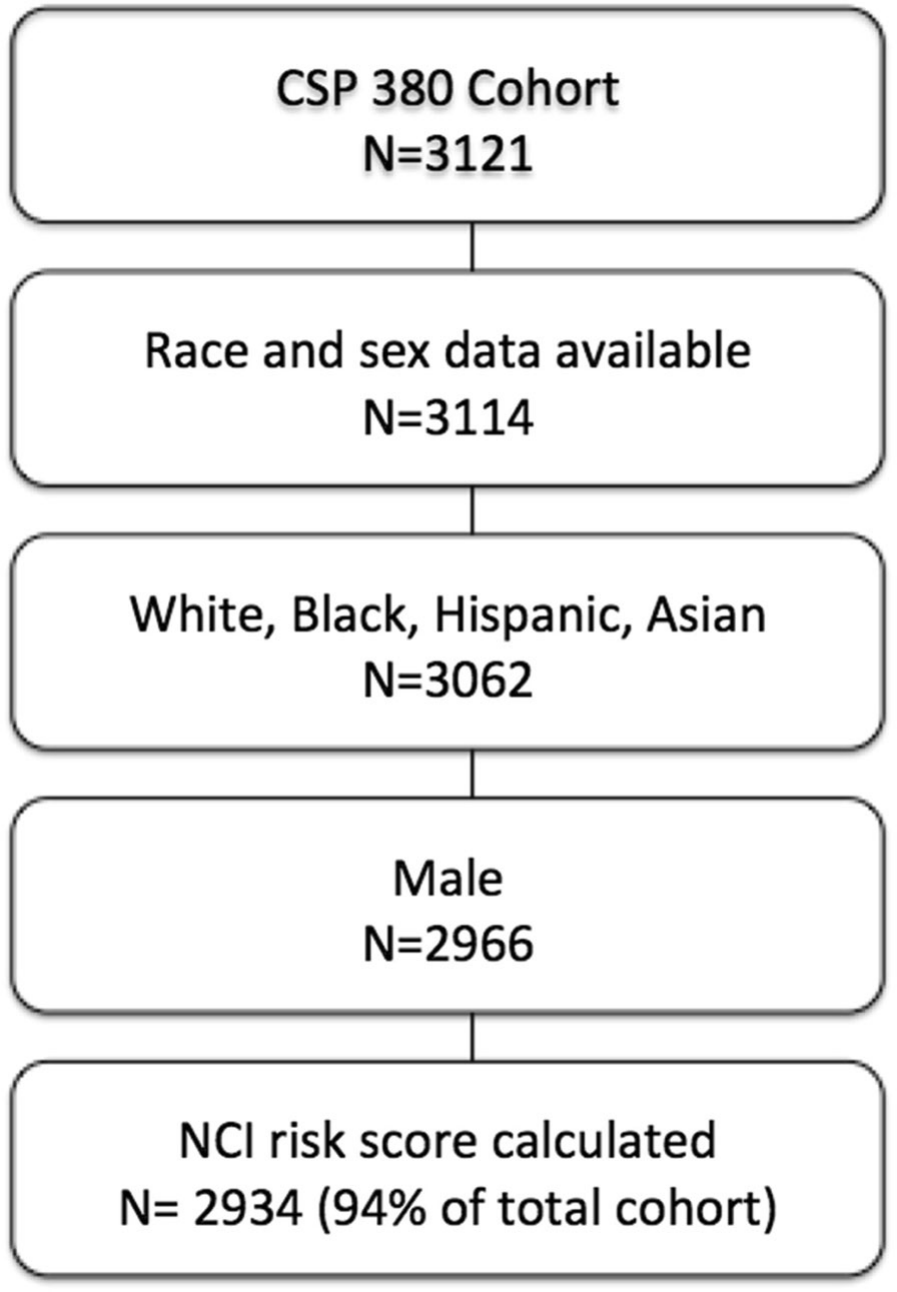
Consort diagram of the study. CSP #380 cohort denotes the Cooperative Studies Program #380 cohort and NCI denotes National Cancer Institute

Veterans were included who had complete race and sex data available, and fit one of four ethnic categories defined in the model. Because veterans were recruited in the 1990s, we removed female participants due to the small number and lack of outcomes needed to apply a separate, NCI female-specific model. Risk scores were computed for 2934 veterans - 94% of the total cohort (Fig. 1).

### Outcomes

Advanced colorectal neoplasia on baseline screening colonoscopy was the primary outcome and was defined by the presence of an adenoma ≥1 cm, villous histology, high-grade dysplasia, or carcinoma. If more than one lesion was present, participants were classified by their most advanced lesion. Centrally trained pathologists blinded to participant information reviewed biopsies at the site of care. Biopsies were then sent for a blinded second review. Discrepancies were resolved by a third referee pathologist.

### Data management

At enrollment and prior to screening colonoscopy, participants completed a validated, detailed questionnaire. Information obtained included dietary habits, physical activity, medical history, medication use, and family history of CRC (Additional files 1 and 2). In this study, we restricted our dataset to CRC risk factors included in the NCI tool. Our data collection was designed for the original CSP #380 study, which aimed to evaluate the use of screening colonoscopy as a colorectal cancer prevention strategy.

Overall, participant data was categorized the same as the variable categories of the NCI tool, with a few minor exceptions. The NCI tool classified regular use of non-steroidal anti-inflammatory drugs (NSAIDs) as three or more doses per week whereas the CSP #380 baseline questionnaire categorized NSAID use as daily or as needed. Participants who responded as daily users of aspirin and/or non-aspirin NSAIDs were designated as “regular users” for this category using the NCI tool. For the vigorous exercise variable in the NCI risk tool, categories were 0 h, 0-2 h, 2-4 h, and greater than 4 h per week. The CSP #380 questionnaire collected this information using two separate questions: “How often does exercise happen and how long does the activity last on average?” Reported exercise was classified as vigorous activity. The average amount of vigorous activity per week was constructed using this coding strategy and number of hours of exercise reported.

### Statistical analysis

**Fig. 2:**
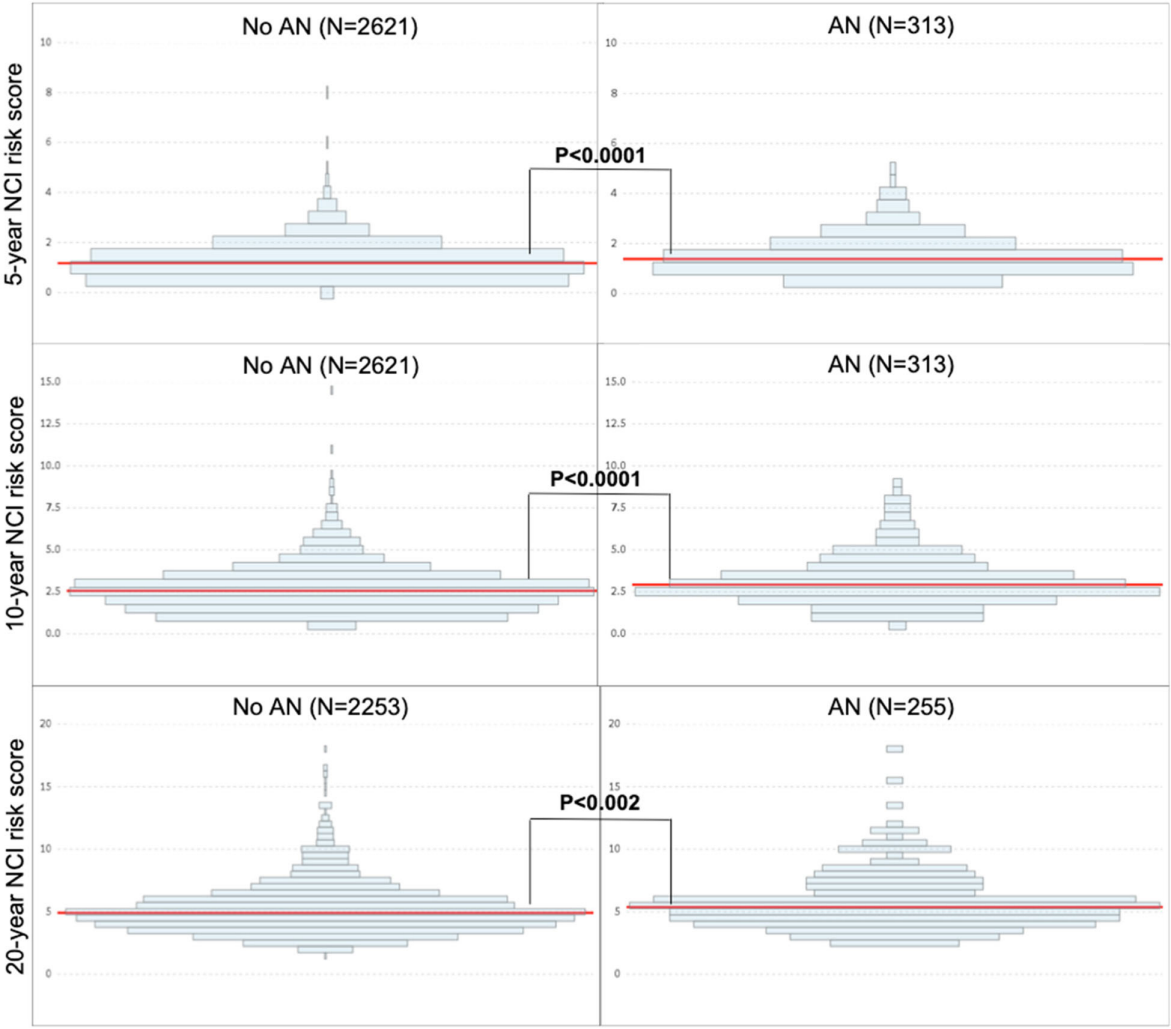
Distribution of NCI CRC Risk Assessment Tool scores for individuals with and without advanced neoplasia. Red horizontal lines represent median risk scores. *P*-values derived from Wilcoxon-rank sum testing of medians between participants without and with advanced neoplasia

We used the NCI CRC Risk-Assessment Tool’s publicly available SAS code to compute individuals’ expected absolute CRC risk at 5, 10, and 20 years (https://dceg.cancer.gov/tools/risk-assessment/ccratsasmacro). We first tabulated the prevalence of variables by risk factor parameters defined by the NCI tool. For each NCI tool time point, we then compared the distribution of risk scores between participants with and without current AN on baseline colonoscopy. Risk scores followed a non-normal distribution and we therefore used the Wilcoxon rank-sum test to test the null hypothesis of no difference in median risk scores among advanced neoplasia cases and non-cases at 5, 10 and 20 years (Fig. 2).

We evaluated the model’s goodness of fit using the area under the receiver-operating characteristics curve (AUC) as derived from a logistic regression model for 5-, 10- and 20-year cut-offs.

We used SAS software for analyses ((version 9.4) SAS Institute Inc., Cary, NC). All analyses were pre-specified and *p*-values are two-sided.

## Results

### Study population

In total, 3121 participants underwent the required screening colonoscopy and completed the questionnaire to be enrolled in the CSP #380 study. Of these, 3114 had race and sex data available. We excluded individuals who could not have a risk score computed (race not applicable in 52 participants and missing in 7 participants). In this veteran population, 100 female veterans were removed due to small sample size or missing data, and lack of AN outcomes necessary to compute a risk score using a separate, female-specific model.

Validation study participants consisted of 2934 male veterans with a median age of 63 (IQR, 57-68) and 15% minorities including 85% white non-Hispanics, 9.7% black non-Hispanics, 4.5% Hispanics and 0.8% Asians (Fig. 1, Table 1).

**Table 1:** Participant baseline characteristics by baseline colonoscopy outcome

Characteristic	All (*N* = 2934)	AN (*N* = 313)	No AN (*N* = 2621)	Chi-Square *p*-value
Age – years				
50-59	986 (33.6%)	58 (18.5%)	928 (35.4%)	*p* < 0.0001
60-69	1398 (47.7%)	183 (58.5%)	1215 (46.4%)	
70-75	550 (18.8%)	72 (23.0%)	478 (18.2%)	
Race				
White (Non-Hispanic)	2493 (85.0%)	273 (87.2%)	2220 (84.7%)	*p* = 0.29
African-American (Non-Hispanic)	285 (9.7%)	22 (7.0%)	263 (10.0%)	
Hispanic	132 (4.5%)	14 (4.5%)	118 (4.5%)	
Asian-American	24 (0.8%)	4 (1.3%)	20 (0.8%)	
Colorectal cancer in 1^o^ relative^a^				
0 or unknown	2526 (86.1%)	256 (81.8%)	2270 (86.6%)	*p* = 0.02
1	378 (12.9%)	50 (16.0%)	328 (12.5%)	
≥ 2	30 (1.0%)	7 (2.2%)	23 (0.9%)	
Vigorous exercise- hrs/wk				
0	1375 (46.9%)	168 (53.7%)	1207 (46.1%)	*p* = 0.01
> 0-2	1370 (46.7%)	122 (39.0%)	1248 (47.6%)	
> 2-4	116 (4.0%)	18 (5.8%)	98 (3.7%)	
> 4	73 (2.5%)	5 (1.6%)	68 (2.6%)	
Regular aspirin/NSAID use				
No	773 (26.4%)	81 (25.9%)	692 (24.6%)	*p* = 0.13
Yes	1581 (53.9%)	157 (50.2%)	1424 (54.3%)	
Do not know	580 (19.8%)	75 (24.0%)	505 (19.3%)	
Smoking –cigs/day				
0 Or Unknown	746 (25.4%)	80 (25.6%)	666 (25.4%)	*p* = 0.68
1-10	402 (13.7%)	36 (11.5%)	366 (14.0%)	
11-20	826 (28.2%)	92 (29.4%)	734 (28.0%)	
> 20	960 (32.7%)	105 (33.6%)	855 (32.6%)	
Vegetable intake -servings/week				
< 5	43 (1.5%)	3 (1.0%)	40 (1.5%)	*p* = 0.43
≥ 5	2891 (98.5%)	310 (99.0%)	2581 (98.5%)	
BMI – kg/m^2^				
< 25	565 (19.3%)	65 (20.8%)	500 (19.1%)	*p* = 0.14
≥ 25 and < 30	1346 (45.9%)	127 (40.6%)	1219 (46.5%)	
≥ 30	1023 (34.9%)	121 (38.7%)	902 (34.4%)	

Number of participants and prevalence are reported unless otherwise denoted. Participants are categorized by baseline colonoscopy outcome

*Abbreviations*: *No.* Number, *1*^*o*^ First degree, *hrs/wk.* Hours per week, *NSAID* Non-steroidal anti-inflammatory drug, *cigs/day* Cigarettes per day, *BMI* Body mass index, *kg/m*^*2*^ Kilograms per meter squared

^a^Participants with unknown family history of CRC or smoking status were assigned to the “0 family members affected” and “none” categories, respectively. Chi-squared tests were used to assess differences in prevalence between CRC cases and non-cases

### Outcomes

In this study, 313 (11%) participants were diagnosed with AN by baseline screening colonoscopy within 6 months of study enrollment. Among these, 27 had CRC present on baseline screening colonoscopy. Table 1 shows the frequency of risk factors used in the NCI Risk Assessment Tool for the CSP #380 cohort study. The distribution of risk factors differed somewhat between participants who did and did not develop AN. Participants who developed AN were more likely to be older, smoke more than one pack of cigarettes daily, have one or more first degree relatives with CRC, and a greater portion had unknown aspirin/NSAID use.

### Risk score distribution by outcome

Individuals with AN were more likely to have a higher risk score than individuals without AN, though there was significant overlap in scores at both time points (Fig. 2). Median risk scores were significantly higher in individuals with AN compared to those without AN at 5 years (1.38 [IQR, 1.03-1.89] vs. 1.18 [IQR, 0.72-1.64]; *p* < 0.001), 10 years (2.92 [IQR, 2.25-3.81] vs. 2.55 [IQR, 1.73-3.32]; *p* < 0.001), and 20 years (5.37 [IQR, 4.29-6.75] vs. 4.91 [IQR, 3.89-6.08]; *p* = 0.002).

### Discriminatory function and tool parameters

**Fig. 3:**
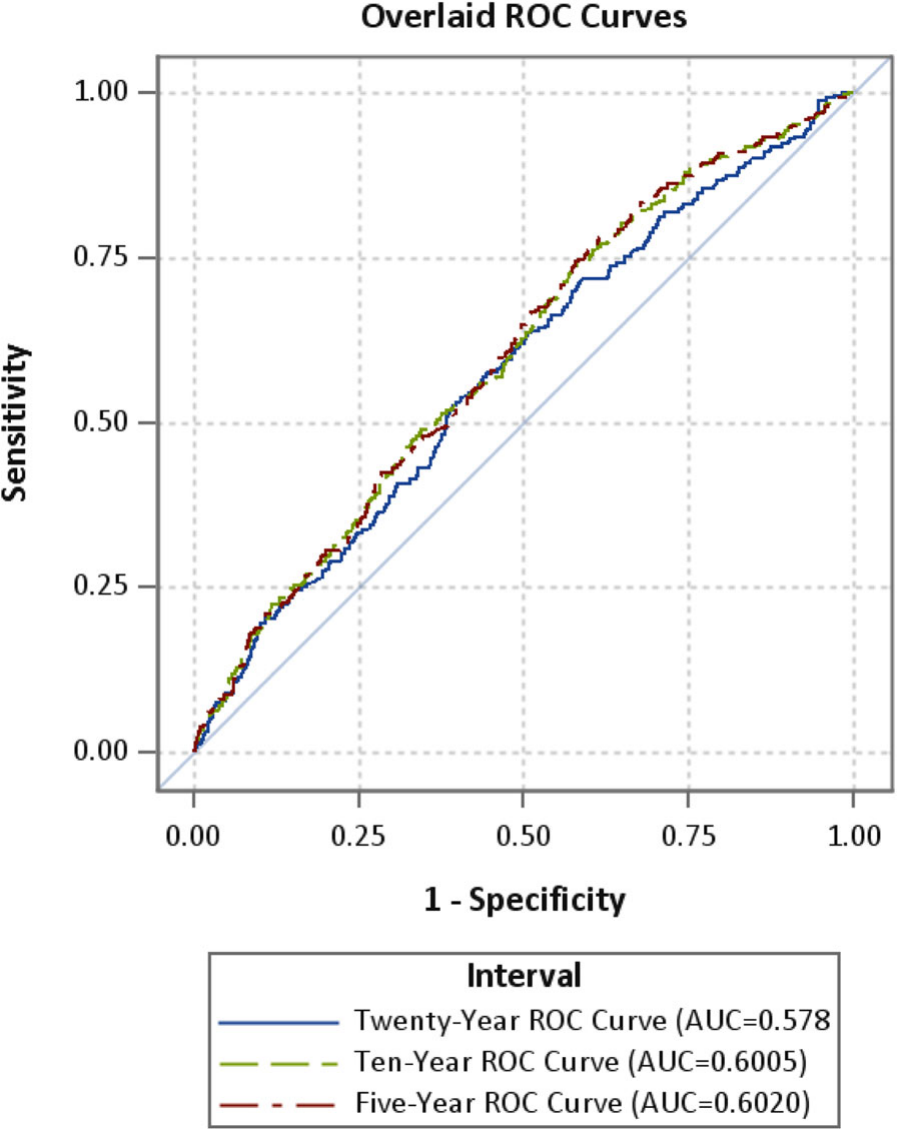
Receiver-operating characteristic (ROC) curves and area under the curve (AUC) statistics for absolute colorectal cancer risk at 5, 10, and 20 years

The AUC for the NCI Risk Assessment Tool was 0.60 (95% CI; 0.57-0.63, *p* < 0.0001) at 5 years, 0.60 (95% CI, 0.57-0.63, *p* < 0.0001) at 10 years and 0.58 (95% CI, 0.54-0.61, *p* < 0.0001) at 20 years, reflecting overall higher predicted risks for participants with baseline advanced neoplasia than those without (Fig. 3).

## Discussion

In this study, we have shown that the NCI Risk Assessment Tool accurately predicts the presence of AN among male veterans undergoing a baseline screening colonoscopy, further supporting recent literature and highlighting its appropriate use in the Veterans Health Administration to inform screening discussions between patients and clinicians.

We evaluated the tool’s discriminatory accuracy and test characteristics, and found that participants with current AN had higher NCI tool risk scores than those without AN, though with significant overlap. ANprevalence increased incrementally with higher risk score, ranging from 6.3-7.5% in the lowest tertile of risk scorers to 12.4-14.2% in the highest risk tertile at the measured timepoints (Table 2). Discriminatory power was moderate using AN prevalence as the outcome and in line with other cancer risk models commonly used in clinical practice, including models for breast cancer (AUC = 0.66) and lung cancer (AUC = 0.61) [13, 14]. Despite modest discriminatory accuracy, there were 2-fold differences in absolute CRC risk between the lowest and highest risk tertiles at the 5 and 10 year time points, suggesting that the tool meets a clinically significant threshold at the population level from which to guide medical decision-making discussions over these time horizons (Table 2).

**Table 2:** Estimated colorectal cancers and prevalence of advanced neoplasia by risk score tertile

AN Outcomes	Risk Tool Tertile	Estimated CRC risk, % (Range)	Prevalence of AN % (95% CI)
5 years	T_1 (n979)_	0.58 (0.72)	6.6.54 (4.99, 8.09)
	T_2 (*n* = 977)_	1.21 (0.58)	11.26 (9.28, 13.24)
	T_3 (*n* = 978)_	2.09 (6.28)	14.21 (12.02, 16.40)
10 years	T_1 (n978)_	1.43 (1.60)	6.34 (4.81, 7.87)
	T_2 (*n* = 978)_	2.59 (0.98)	11.25 (9.27, 13.23)
	T_3 (*n* = 978)_	4.18 (11.24)	14.42 (12.22, 16.62)
20 years	T_1 (*n* = 836)_	3.42 (2.61)	7.54 (5.75, 9.33)
	T_2 (*n* = 836)_	4.95 (1.39)	10.53 (8.45, 12.61)
	T_3 (*n* = 836)_	7.48 (12.34)	12.44 (10.2, 14.68)

*T* Tertile and is ranked in order of low (T_1_) to high (T_3_) risk score, *N* Number, *CI* Confidence interval

In addition, C-statistics are nearly identical to those reported in other external validations of the NCI tool for both baseline AN on screening colonoscopy [8, 9] and invasive CRC in population-based prospective cohorts with a time horizon of 5 (UK Biobank, AUC = 0.60), 8 (NIH-AARP, AUC = 0.60) and 10 years (EPIC, AUC = 0.61) [6, 15]. A retrospective study by Tariq et al. included 749 ethnically and gender diverse participants (91% African American and Asian, 58% female) and revealed an AUC of 0.62. This study was limited to a single center retrospective experience and included patients undergoing surveillance colonoscopy in addition to screening colonoscopy. A recent smaller study by Ladabaum et al. was performed in another ethnically and racially diverse group of participants undergoing screening colonoscopy, whereby an 11 % prevalence of baseline AN was observed, similar to ours [8]. The overall AUC was 0.62, while for sex-specific analyses, it was slightly lower at 0.59, for women, and slightly higher for men at 0.63, suggesting that risk prediction is slightly diminished for women. Alternately, there was no difference in discriminatory accuracy in an external validation by Park et all for future CRC risk prediction [6]. Imperiale and colleagues performed a similar validation study with participants recruited from multiple health systems throughout the country [9] with similarly drawn conclusions that the NCI tool has dual risk prediction capabilities. Together, our study provides further evidence for its clinical use in veterans, who account for over 18 million U.S. citizens at present [16], 9 million of whom currently access the VA for healthcare, and many of whom have unique exposures that may confer additional cancer risk [10, 17].

Until recently, the NCI CRC Risk Assessment Tool was one of the only externally validated CRC risk models available for use in the primary CRC prevention setting. In 2019, Smith and colleagues systematically identified published CRC risk prediction models and externally validated them using two large population-based cohorts. Overall, models required between 3 and 13 variables, and moderate-to good AUCs up to 0.70 were reported, thereby broadening the pool of available risk prediction models to choose from in clinical settings [15], based on the available clinical variables.

A fundamental challenge for CRC prevention is screening adoption. In the U.S., current CRC screening rates are 67% [18], while among veterans the screening rate is 76% [19]. Among veterans, screening rates are even higher for patients with primary health insurance coverage through the VA or military compared to Veterans with private coverage, Medicare or Medicaid. And so, the VA health system may offer a unique, closed health system environment from which to evaluate strategies that continue to impact screening uptake. Utilizing risk prediction tools such as the NCI CRC Risk Assessment Tool in clinical practice may help personalize care by providing individuals with a better understanding of personal risk for CRC, and thus encourage adherence to screening recommendations. Indeed, it has been shown that CRC screening uptake is increased when a choice between invasive and non-invasive screening modalities is offered [20]. Among individuals determined to be low risk, incorporating a risk assessment into this decision could further increase the uptake of CRC screening as these individuals may be more confident in deciding to pursue more readily available, non-invasive screening modalities such as Fecal Immunochemical Testing (FIT). Alternatively, among individuals determined to be at higher risk using the NCI tool, a screening colonoscopy may be of more utility, as FIT was recently shown to have low sensitivity for advanced adenoma detection as a single application test [21]. Finally, a risk prediction tool based on a composite summary of demographic, clinical, and lifestyle risk factors could be routinely calculated in the electronic health record by information obtained prior to the primary care provider’s visit, similar to a cardiovascular risk score, which could then prompt discussion of the risk factors predominately driving these scores to motivate lifestyle interventions and changes by the patient.

American Cancer Society guidelines suggest CRC screening for patients 45 - 75 years old [22], while current National Comprehensive Cancer Network and Multi-society Task Force Guidelines recommend screening patients 50 - 75 years old, and for some higher risk patients who are 76-85 years old. Additionally, these guidelines suggest considering the potential benefits of CRC screening and balancing this with possible harms, including life-limiting co-morbidities, for which invasive testing may be unsafe or unlikely to provide a net benefit [23, 24]. At a population level, it is known that screening colonoscopy reduces advanced colorectal neoplasia, though it remains unknown whether there is a CRC mortality benefit [25]. At present there is a large, randomized controlled trial across the VA health system of Colonoscopy versus Fecal Immunochemical Test in Reducing Mortality from CRC (CONFIRM Trial) that aims to address this uncertainty. Meanwhile, for individual patients, up to 85% will have no neoplasia on screening colonoscopy [26], highlighting that a majority of patients screened will not personally benefit while all are exposed to the harms associated with colonoscopy. In clinical practice, we believe the NCI tool could help estimate the likelihood that a screened individual will directly benefit from undergoing screening colonoscopy and may best be used to frame a patient-centered discussion of when and whether to undergo screening colonoscopy. Alternatively, opting for a less invasive screening modality may be more appropriate after considering medical conditions and other well-described CRC risk factors that may influence the safety or utility of undergoing colonoscopic screening. This notion is supported in a study by Chiu et al., where they found that use of The Asia-Pacific Colorectal Screening risk tool correctly triaged 95% of participants with CRC and 71% of those with AN to undergo colonoscopy as opposed to FIT [27]. Thus, risk prediction tools may help reduce the indiscriminate use of costly, low yield, invasive procedures in those with minimal CRC risk.

There are limitations to this study. The CSP #380 cohort was made up of veteran participants recruited in the 1990s, were therefore predominantly men, and we were unable to assess the tool’s utility in women. While the CSP #380 cohort does represent the current make-up of U.S. veterans, low representation of women is a common shortcoming for VA-based research. This will become increasingly important to address as the veteran workforce is projected to double in the percentage of women over the next 30 years [28]. We additionally did not have measurements of waist circumference, which would have allowed us to compare the NCI tool to a similar model incorporating five clinical risk factors for CRC by Imperiale and colleagues, which has also been externally validated [29]. Given that this is a screening population without prior endoscopic procedures, we were unable to determine the ability of the NCI tool to quantify risk at subsequent exams or the utility of repeating screening or surveillance. Additionally, these risk prediction tools are only as reliable as the input data, and so it is possible that information regarding participants’ diet, physical activity, family history, or medication adherence may be imperfect. Finally, those without AN have scores that substantially overlapped with those who had AN, which may pose a challenge to accurately discriminating between risk groups in routine clinical practice. Therefore, we would caution against using this tool as the only discussion point between patients and clinicians on the utility and modality of colorectal cancer screening. Certainly, patient preference, comorbidities, life expectancy, cost, and capacity of a healthcare system are important additional factors to consider. It remains to be seen if expanding these tools with genetic and genomic information will improve risk prediction, screening uptake, and CRC mortality.

## Conclusions

In summary, we demonstrated that a simple risk assessment tool performs well in discerning individual risk for AN. In doing so, the tool may assist in assessing the risks and benefits of screening and the method by which to do so (colonoscopy versus a non-invasive modality) in the context of aging and emerging comorbidities. Lower risk individuals could elect to undergo less invasive screening or to forego it altogether.

## Supplementary information

**Additional file 1.** VA Cooperative Study #380 Clinic Survey Form.

**Additional file 2.** VA Cooperative Study #380 Medical History Form.

## Data Availability

The datasets generated and/or analyzed during the current study are available from the corresponding author on reasonable request. Investigators (non-VA and VA) are invited to submit data and specimen requests for the Cooperative Studies Program 380 Cohort. The CSP 380 data dictionary is publicly available: https://www.research.va.gov/programs/csp/cspec/datadictionary_csp380.html#ColaLowCal. The National Cancer Institute Risk Assessment Tool is publicly available: https://ccrisktool.cancer.gov/.

## Abbreviations

AN: Advanced neoplasia
BMI: Body mass index
CRC: Colorectal cancer
CSP: Cooperative Studies Program
NCI: National Cancer Institute
NSAID: Non-steroidal anti-inflammatory drug
SEER: Surveillance and Epidemiology and End Results
VA: Veterans Affairs
