# Supplementary material for: Advanced neoplasia in Veterans at screening colonoscopy using the National Cancer Institute Risk Assessment Tool
Source: BMC Cancer. 2019 Nov 12;19:1097. doi: 10.1186/s12885-019-6204-1 (PMC6852743; doi:10.1186/s12885-019-6204-1)
Supplement: Supplementary file 1 — Additional file 1. VA Cooperative Study #380 Clinic Survey Form. [file 12885_2019_6204_MOESM1_ESM.pdf]

VA COOPERATIVE STUDY #380 FORM 01

"Risk Factors for Large (≥1 cm) Colonic Adenomas"

CLINIC SURVEY FORM

Medical Center Name ______________________ Medical Center No. _ _ _

Patient Name ______________________ Patient No. _ _ _ _

Form Completed By ______________________ Date _ _ - _ _ - _ _ (Mo Day Yr)

Patient's Address: ______________________

Street

____

City State Zip

Telephone: (_ _ _) _ _ _ - _ _ _ _

1. DATE OF BIRTH . . . . Mo _ _ Day _ _ Yr _ _

2. SEX (1=Male, 2=Female) . . . . ___

3. RACE . . . . ___
   1 = American Indian or Alaskan Native
   2 = Asian or Pacific Islander
   3 = Black, not of Hispanic origin
   4 = Hispanic
   5 = White, not of Hispanic origin

4. MARITAL STATUS . . . . ___
   1 = Married
   2 = Remarried
   3 = Widowed
   4 = Separated
   5 = Divorced
   6 = Never married

5. HIGHEST LEVEL OF EDUCATION ATTAINED . . . . ___
   1 = Under 7 years schooling
   2 = Junior high school (7th-9th grade)
   3 = Partial high school (10th-11th grade)
   4 = High school graduate
   5 = Partial college training
   6 = Standard college/university graduate
   7 = Completed graduate/professional training

VA FORM 10-20955(NR)a
OCTOBER 1993

6. USUAL KIND OF WORK DURING MOST OF YOUR WORKING LIFE . . . . ___
   1 = Never gainfully employed
   2 = Unskilled employee
   3 = Machine operator, semi-skilled employee
   4 = Skilled manual employee
   5 = Clerical or sales worker, technician, owner of small business
   6 = Administrative personnel, owner of small independent business, minor professional
   7 = Business manager of large concern, proprietor of medium-sized business, lesser professional
   8 = Higher executive, proprietor of large concern, major professional

7. USUAL EMPLOYMENT PATTERN DURING THE PAST 3 YEARS . . . . ___
   1 = Full-time (40 hours/week)
   2 = Part-time (regular hours)
   3 = Part-time (irregular workday)
   4 = Student
   5 = Military service
   6 = Retired/disability
   7 = Unemployed

PHYSICAL ACTIVITY

8. Compared with other persons of your age and sex, how physically active do you consider yourself (include activity at work, around the home, and in recreation)? (ENTER ONE NUMBER FROM BELOW) . . . . ___

1	2	3	4	5
Much Less Active	Somewhat Less Active	About the Same	Somewhat More Active	Much More Active

9. Considering both occupational and leisure-time activity, do you engage in any vigorous activities? ("Vigorous" activities are those that cause you to perspire or to breathe heavily.) (1=Yes, 2=No) . . . . ___

   IF YES:

   A. How often does this occur? . . . . ___
      1=More than once daily
      2=Once daily
      3=Four to six times a week
      4=One to three times a week
      5=One to three times a month
      6=Less than once per month
      7=Don't know

   B. On the average, how long does the activity last each time? . __ __ hours __ __ minutes

SMOKING HISTORY

10. Do you smoke cigarettes daily now? (1=Yes, 2=No) . . . . ___

IF YES:

A. How many cigarettes do you smoke per day? . . . . ___ ___ ___

B. How many years have you been smoking cigarettes? (Go to Question 12.) . . . . . ___ ___

11. Were you a previous cigarette smoker? (1=Yes, 2=No) . . . . ___

IF YES:

A. How many years ago did you quit smoking cigarettes? . . . . ___ ___

B. How many years did you smoke cigarettes? . . . . ___ ___

C. How many cigarettes did you smoke per day? . . . . ___ ___ ___

ALCOHOL

12. Do you currently drink alcoholic beverages? (1=Yes, 2=No) . . . . ___

IF NO, GO TO QUESTION 13.

IF YES, INDICATE DRINKS PER WEEK BELOW:

A. Beer (1 bottle or can) . . . . ___ ___ ___

B. Wine (1 glass) . . . . ___ ___ ___

C. Liquor (1 shot) . . . . ___ ___ ___

13. Did you drink alcoholic beverages in the past? (1=Yes, 2=No) . . . . ___

IF NO, GO TO QUESTION 14.

IF YES, INDICATE DRINKS PER WEEK BELOW:

A. Beer (1 bottle or can) . . . . ___ ___ ___

B. Wine (1 glass) . . . . ___ ___ ___

C. Liquor (1 shot) . . . . ___ ___ ___

HAVE YOU EVER HAD ONE OR MORE OF THE COLON EXAMINATIONS LISTED BELOW:

14. Sigmoidoscopy (tube with a light inserted into rectum and colon) (1=Yes, 2=No) . . . . ____
    A. If Yes, give date of most recent examination . . . . Mo __ __ Yr __ __

15. Colonoscopy (tube with a light inserted into colon after you are given medicine to make you sleepy) (1=Yes, 2=No) . . . . ____
    A. If Yes, give date of most recent examination . . . . Mo __ __ Yr __ __

16. Barium Enema (1=Yes, 2=No) . . . . ____
    A. If Yes, give date of most recent examination . . . . Mo __ __ Yr __ __

17. HAS ANY MEMBER OF YOUR FAMILY HAD COLON CANCER? (1=Yes, 2=No) . . . ____

    IF YES, PLEASE INDICATE WHICH FAMILY MEMBERS:
    A. Parents (1=Yes, 2=No) . . . . ____
    B. Brothers or sisters (1=Yes, 2=No) . . . . ____
    C. Aunts or uncles (1=Yes, 2=No) . . . . ____
    D. Grandparents (1=Yes, 2=No) . . . . ____
    E. Cousins (1=Yes, 2=No) . . . . ____
    F. Other distant relatives (1=Yes, 2=No) . . . . ____

18. HAS YOUR GALLBLADDER BEEN REMOVED? (1=Yes, 2=No) . . . . ____
    A. If Yes, give date . . . . Mo __ __ Yr __ __

19. ARE YOU TAKING COUMADIN/WARFARIN? (1=Yes, 2=No) . . . . ____

20. HAVE YOU MADE A MAJOR CHANGE IN YOUR DIET IN THE LAST FIVE YEARS? (1=Yes, 2=No) . . . . ____
    A. If Yes, please describe: ____

    ____

    ____

VA FORM 10-20955(NR)a
OCTOBER 1993

 Medical Center No. __ __ __ Patient No. __ __ __ __

21. PLEASE INDICATE HOW FREQUENTLY YOU EAT EACH OF THE FOLLOWING ITEMS LISTED BELOW.

	1<br>Never	2<br>1-3 times per month	3<br>1 time per week	4<br>2-4 times per week	5<br>5-6 times per week	6<br>1 time per day	7<br>2-3 times per day	8<br>4-5 times per day
A. Processed Meats (sausage, salami, bologna, etc.)								
B. Beef, Pork, Lamb as a main dish (steak, roast, ham, etc.)								
C. Beef, Pork, Lamb as part of a sandwich or mixed dish (stew, casserole, lasagna)								
D. Chicken or Turkey with Skin								
E. Chicken or Turkey without Skin								
F. Fish								
G. Fruit Servings								
H. Vegetable Servings								

P.I. Signature ______________________________
