# Supplementary material for: Advanced neoplasia in Veterans at screening colonoscopy using the National Cancer Institute Risk Assessment Tool
Source: BMC Cancer. 2019 Nov 12;19:1097. doi: 10.1186/s12885-019-6204-1 (PMC6852743; doi:10.1186/s12885-019-6204-1)
Supplement: Supplementary file 2 — Additional file 2. VA Cooperative Study #380 Medical History Form. [file 12885_2019_6204_MOESM2_ESM.pdf]

VA COOPERATIVE STUDY #380

"Risk Factors for Large (≥1 cm) Colonic Adenomas"

FORM 04

MEDICAL HISTORY

Medical Center Name ______________________ Medical Center No. ___ ___ ___

Patient Name ______________________ Patient No. ___ ___ ___ ___

Form Completed By ______________________ Date ___ ___ - ___ ___ - ___ ___ (Mo Day Yr)

A. MEDICAL HISTORY

CODE: 1=YES 2=NO

1. Did a doctor ever tell you if you had:
   - A. Hypertension . . . . ___
   - B. Diabetes . . . . ___
   - C. Emphysema . . . . ___
   - D. Chronic bronchitis . . . . ___
   - E. Coronary heart disease . . . . ___
   - F. A stroke or transient ischemic attack (TIA) . . . . ___
   - G. Chronic pancreatitis . . . . ___
   - H. Hepatitis . . . . ___
   - I. Hemorrhoids . . . . ___
   - J. Kidney disease . . . . ___
   - K. Gallstones . . . . ___
   - L. Peptic ulcer . . . . ___
   - M. Arthritis . . . . ___
   - N. Diverticulitis or diverticulosis . . . . ___
   - O. High levels of cholesterol . . . . ___
   - P. Any disease of the thyroid gland . . . . ___

2. Did a doctor ever tell you if you had any type of cancer or malignant tumor? . . . . ___
   - A. If Yes, describe ______________________

3. List any other active medical problems:
   - A. ______________________
   - B. ______________________
   - C. ______________________
   - D. ______________________

1=YES 2=NO

4. History of bleeding tendency? . . . . ___
   - A. If Yes, describe ______________________

5. History of rheumatic fever? . . . . ___

B. PRIOR SURGERY

6. Cholecystectomy (1=Yes, 2=No) .... ___
   A. If Yes, give date .... Mo _ _ Yr _ _

7. List other major surgeries:
   A. ______
   B. ______
   C. ______
   D. ______

C. NON-STEROIDAL ANTI-INFLAMMATORY DRUGS

		1=YES 2=NO	If Yes:	How Often? 1=Daily 2=As Needed	Duration (Years)
8.	Aspirin	___		___	_ _
9.	Sulindac	___		___	_ _
10.	Ibuprofen	___		___	_ _
11.	Indocin	___		___	_ _
12.	Feldene (Piroxicam)	___		___	_ _
13.	Are you taking anything for pain?	___		___	_ _

A. If Yes, list: ______

D. GI HISTORY

1=YES 2=NO

14. History of heartburn, esophagitis .... ___
15. History of dyspepsia (burning stomach pain relieved with antacids, food) .... ___
16. Prior peptic ulcer disease .... ___
17. History of prior upper GI endoscopy .... ___
    A. If Yes, results ______

18. Quality of bowel movement .... ___
    1=Hard like rabbit pellets 3=Soft but firm
    2=Moderately hard 4=Soft and loose

E. **ACTIVITY** (see Physical Activity Chart)

[illegible] On a typical <u>WEEKDAY</u> during the past year, how many hours did you spend doing the following activities? The total should equal 24 hours.

A. Sleeping	__ __
B. Very light activity	__ __
C. Light activity	__ __
D. Moderate activity	__ __
E. Heavy activity	__ __
TOTAL	2 4

20. On a typical <u>WEEKEND day</u> during the past year, how many hours did you spend doing the following activities? The total should equal 24 hours.

A. Sleeping	__ __
B. Very light activity	__ __
C. Light activity	__ __
D. Moderate activity	__ __
E. Heavy activity	__ __
TOTAL	2 4

21. Do you have any difficulty climbing a flight of stairs or walking eight blocks due to a physical impairment? (1=Yes, 2=No) . . . . ____

22. How many flights of stairs (not steps) do you climb daily? . . . . __ __ __

23. <u>During the past year</u>, what was your <u>average total time per week</u> at each activity:

Activity	HOURS	MINS.
A. Watching TV or VCR	__ __ __	__ __
B. Sitting or driving in a car, bus or train	__ __ __	__ __
C. Sitting at work	__ __ __	__ __
D. Sitting at home (other than watching TV or VCR)	__ __ __	__ __
E. Walking or hiking outdoors (including walking at golf)	__ __ __	__ __
F. Jogging (slower than 10 minutes/mile)	__ __ __	__ __
G. Running (10 minutes/mile or faster)	__ __ __	__ __
H. Bicycling (including stationary machine)	__ __ __	__ __
I. Lap swimming	__ __ __	__ __
J. Tennis	__ __ __	__ __
K. Squash or Racquetball	__ __ __	__ __
L. Calisthenics or Rowing	__ __ __	__ __
M. Weightlifting or Nautilus	__ __ __	__ __
N. Heavy outdoor work (e.g., digging, chopping)	__ __ __	__ __

F. FAMILY HISTORY

These questions ask about a history of several illnesses in your first-degree relatives; that is, your natural parents, full brothers and sisters, and children. List each first-degree relative that had colon or rectal cancer, colon or rectal polyps, or any other illnesses; their age when they first developed the illness (as closely as you can recall) or their date of death (if deceased). Code each illness this relative had and age of onset.

RELATIVE CODE<br>1=Father (natural)<br>2=Mother (natural)<br>3=Brother<br>4=Sister<br>5=Children	CURRENT AGE (if still living)	AGE AT DEATH (if deceased)	CODE EACH DIFFERENT ILLNESS THIS RELATIVE HAD AND AGE OF ONSET.<br>CODES: 01=Colon or rectal cancer; 02=Colon or rectal polyps; 03=Breast cancer; 04=Lung cancer; 05=Ovarian cancer; 06=ENT cancer; 07=Lymphoma; 08=Leukemia; 09=Esophageal cancer; 10=Gastric cancer; 11=Other cancer<br>CODE	AGE	CODE	AGE	CODE	AGE	CODE	AGE
24. ___	__ __	__ __	__ __	__ __	__ __	__ __	__ __	__ __	__ __	__ __
25. ___	__ __	__ __	__ __	__ __	__ __	__ __	__ __	__ __	__ __	__ __
26. ___	__ __	__ __	__ __	__ __	__ __	__ __	__ __	__ __	__ __	__ __
27. ___	__ __	__ __	__ __	__ __	__ __	__ __	__ __	__ __	__ __	__ __
28. ___	__ __	__ __	__ __	__ __	__ __	__ __	__ __	__ __	__ __	__ __
29. ___	__ __	__ __	__ __	__ __	__ __	__ __	__ __	__ __	__ __	__ __
30. ___	__ __	__ __	__ __	__ __	__ __	__ __	__ __	__ __	__ __	__ __
31. ___	__ __	__ __	__ __	__ __	__ __	__ __	__ __	__ __	__ __	__ __
32. ___	__ __	__ __	__ __	__ __	__ __	__ __	__ __	__ __	__ __	__ __
33. ___	__ __	__ __	__ __	__ __	__ __	__ __	__ __	__ __	__ __	__ __

P.I. Signature ____________________

PHYSICAL ACTIVITY CHAR[illegible]R FORM 04 (Questions 19 and 20)

TYPES OF ACTIVITIES

	Self-Care of Home Maintenance	Occupational	Recreational/Exercise
VERY LIGHT	Washing, shaving, dressing, desk work, washing dishes, driving auto	A job that requires sitting. Walking and standing are required only occasionally.<br>Examples:<br>Editor<br>Manager (insurance office)<br>Clerical or assembling work	Shuffleboard, watching TV, reading, horseshoes, billards, golf (cart), walking
LIGHT	Light housework	A job that involves sitting, walking or standing with a degree of pushing or pulling of leg or arm controls. Lifting 20 lbs. maximum and carrying objects weighing up to 25 lbs.<br>Examples:<br>Surveyor<br>Stocking shelves<br>Light welding<br>Light carpentry<br>Auto repair	Level bicycling<br>Light calisthenics<br>Sailing<br>Table tennis<br>Dancing (social and square)<br>Golf (walking)
MODERATE	Home repairs<br>General gardening<br>Vacuuming<br>Raking<br>Lawn mowing<br>Climbing stairs (slowly)<br>Carrying objects (30-60 lbs.)	Lifting 50 lbs. maximum with frequent lifting and or carrying of objects up to 50 lbs.<br>Examples:<br>Laundry operator<br>Carpentry (exterior home building)<br>Shoveling dirt<br>Mechanics<br>Fire inspector	Walking (3-4 mph)<br>Bowling<br>Badminton<br>Tennis (singles)<br>Basketball<br>Football<br>Horseback riding
HEAVY	Sawing wood<br>Heavy shoveling<br>Climbing stairs (moderate speed)<br>Carrying objects (60-90 lbs.)<br>Heavy yard work	A job that requires lifting of 100 lbs. maximum and/or carrying of objects weighing up to 50 lbs.<br>Examples:<br>Tending furnace<br>Digging ditches<br>Pick and shovel	Jogging (5 mph)<br>Aerobics<br>Canoeing<br>Mountain climbing<br>Rowing machine
